# Severe Psychotic Disorder as the Main Manifestation of Adrenal Insufficiency

**DOI:** 10.1155/2015/512430

**Published:** 2015-04-12

**Authors:** Julia de Lima Farah, Carolina Villar Lauand, Lucas Chequi, Enrico Fortunato, Felipe Pasqualino, Luis Henrique Bignotto, Rafael Loch Batista, Ivan Aprahamian

**Affiliations:** ^1^Department of Internal Medicine, Faculty of Medicine of Jundiaí, Jundiaí, Brazil; ^2^Hospital das Clínicas da Faculdade de Medicina da Universidade de São Paulo (USP), Institute of Psychiatry, Dr. Ovidio Pires de Campos, 785-1 Andar, Ala Sul, Sala Chefias Médicas, 05403-010 São Paulo, SP, Brazil

## Abstract

We describe a case of severe psychotic disorder as the only manifestation of primary adrenal insufficiency. A 63-year-old man presented with psychotic symptoms without any prior psychiatric history. During the clinical and laboratorial investigation, exams revealed a normovolemic hyponatremia. The patient showed no other clinical signs or symptoms compatible with adrenal insufficiency but displayed very high ACTH and low serum cortisol concentrations. Brain magnetic resonance imaging showed no significant changes, including the pituitary gland. The patient was initially treated with intravenous corticosteroids, resulting in rapid remission of the psychotic symptoms. The association between adrenal insufficiency and neuropsychiatric symptoms is rare but these symptoms can often be the first clinical presentation of the disease.

## 1. Introduction

Adrenal insufficiency is a potential life-threatening disease resulting from primary (adrenal glands) or secondary and tertiary (hypothalamic-pituitary axis) failure of glucocorticoids action or production with or without impairment also in mineralocorticoids and adrenal androgens [1]. It is a relatively rare medical condition, but its prevalence has increased in the last 35 years [2]. According to an expressive registry the most frequent etiology is autoimmune adrenalitis [1, 2]. Typical or cardinal symptoms of the disease include fatigue, weakness, abdominal pain, nausea and vomit, anorexia, weight loss, myalgia, arthralgia, salt craving orthostatic hypotension, and skin hyperpigmentation (primary adrenal failure) [1].

Although uncommon, neuropsychiatric manifestations, such as depressive symptoms, irritability, sleep disorders, apathy, cognitive impairment, delusions, and hallucinations, can also be seen during adrenal insufficiency [3, 4]. Psychiatric symptoms are rarely seen as the initial and especially isolated clinical manifestations of the disease (4). Rather, they usually accompany the cardinal symptoms of adrenal insufficiency and are more correlated with disease severity. Despite the importance of this issue, many physicians, including psychiatrists and endocrinologists, are not aware of the correlation between these disorders. We present a case report of adrenal insufficiency manifested by a severe psychotic syndrome as the main manifestation of this disorder.

## 2. Case Report

A Caucasian 63-year-old man presented a five-month history of progressive depressive symptoms with sadness, anhedonia, asthenia, hyporexia, insomnia, limb weakness, and psychotic symptoms. He was a former heavy smoker and had been diagnosed with chronic occupational lung disease (silicosis). After a specialized psychiatric consultation, the patient started taking fluoxetine, risperidone, and nitrazepam. After a short period of time, the patient withdrew his medication by his own because of worsening of the limb weakness. The patient became severely paranoid with persecutory beliefs, delusions, an infantile speech, progressive social isolation, fear of leaving his home, periods of mental confusion and disorientation, and sporadic nausea and vomits. A few days before hospital admission, the patient almost stopped fluid intake and evolved with syncope.

On the physical examination upon admission, the patient was in regular general health but moderately dehydrated, with slowed peripheral perfusion, arterial blood pressure of 80/50 mmHg, pulse rate of 84/min, blood glucose concentration of 105 mg/dL, and a respiratory rate of 18/min. On neurological examination the following was observed: sleepiness, obeying verbal commands, being disorientated about his self, time, and place, and hyporeflexia over arms and legs. Respiratory examination revealed diffuse rhonchi and base crackles with oxygen saturation of 91%. Cardiovascular and abdominal examination was unremarkable. No cyanosis, jaundice, edema, fever, or skin hyperpigmentation was observed.

**Figure 1:**
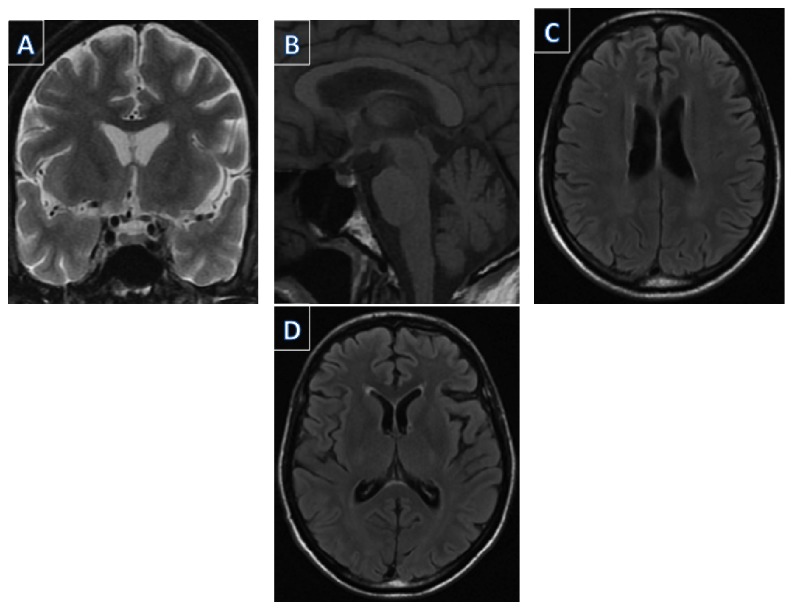
Magnetic resonance imaging of the brain was performed without significant alterations.

Laboratorial exams were within reference limits with the exception of severe hyponatremia (111 mEq/dL) and hyperkalemia (5.8 mEq/dL; Table 1). Chest tomography evidenced fibrosis, bronchiectasis, and findings compatible with pulmonary obstructive disease. Intravenous fluids improved the patient's clinical condition and arterial pressure. However, mental confusion, delusional thoughts, echolalia, infantile speech, and psychomotor agitation persisted despite the correction of electrolyte disturbances. The psychotic symptoms were also refractory to psychopharmacological treatment. Magnetic resonance imaging of the brain was performed without significant alterations (Figure 1). Further investigations pointed out extremely elevated concentration of adrenocorticotropic hormone (ACTH) and a very low level of cortisol, confirming adrenal insufficiency (Table 1).

**Figure 2:**
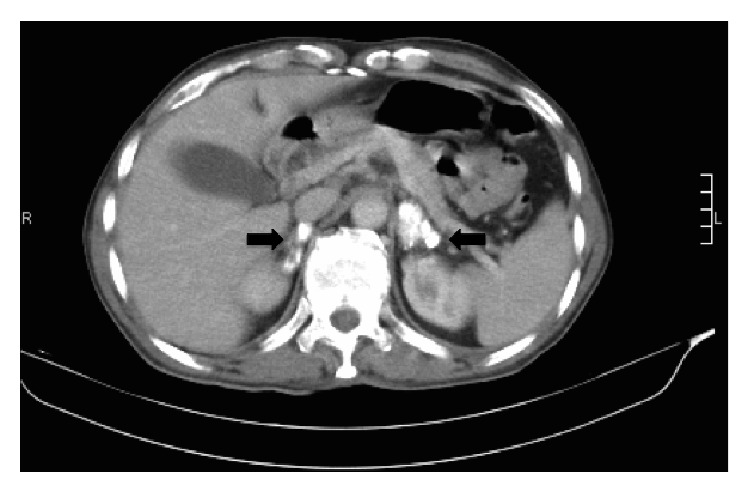
Abdominal tomography revealed bilateral calcifications of both adrenal glands.

Etiological evaluation was performed. Abdominal tomography revealed bilateral calcifications of both adrenal glands (Figure 2). These findings suggested an infectious process of granulomatous nature, with the first hypothesis of paracoccidioidomycosis. A negative serology for blastomycosis and a normal bronchoalveolar lavage rule out the fungal infection. Tuberculosis was also ruled out. During treatment with intravenous hydrocortisone the patient improved significantly with regard to the psychotic and confusional symptoms at the third day. Patient remained calm, cooperative, and oriented in time and space, without depressive symptoms or psychosis. Patient was discharged with prednisone daily after twenty-three days of hospital care to a followup at the endocrinology department outpatient care.

## 3. Discussion

This case report illustrates an atypical clinical presentation of adrenal insufficiency, formally known as Addison's disease. The severe psychotic symptomatology that was resistant to antipsychotic drugs ceased after three days of hydrocortisone treatment. This is a rare case in which a psychiatric disorder was the main manifestation of long-term adrenal insufficiency. Additionally, our patient did not present with a severe clinical manifestation and cardinal signs of this metabolic disorder, such as an Addisonian crisis. Psychiatric manifestations of Addison's disease were first reported by Klipel in 1899 defined as “Addisonian encephalopathy” [5]. A few case series (*n* = 25) were published during the 1940s and 1950s revealing a high association (i.e., 64–85%) between psychiatric disorders and Addison's disease 6–8. However, nowadays this association receives little attention.

Most case studies of Addison's disease with psychiatric manifestations depict that men are slightly more affected than women and this symptomatology usually starts before the diagnosis and treatment of adrenal insufficiency [6–8]. Depression is the most common disorder associated with adrenal insufficiency, with usually mild mood symptoms, a decrease in motivation, and altered behavior [7]. Psychosis, delirium, catatonia, memory impairment, and disorientation are less frequently seen [9]. Psychosis is more correlated with Addisonian crisis and severe manifestations of the disease, and only nine case reports correlating a psychotic manifestation with the initial clinical picture of adrenal insufficiency have been published in the current literature [8].

The etiology of psychiatric disorders in Addison's disease is not well explained. Several theories were elaborated based on a few case reports. Hyponatremia is associated with encephalopathy and brain swelling and could produce disorders of consciousness, memory, and thinking [1]. Glucocorticoid receptors are present in the brain, especially at the hippocampi [9]. Lower glucocorticoid stimulation could lead to memory impairment and frontal circuit dysfunction with decreased executive function, processing speed, reasoning, and thinking [10]. A decrease in cerebral glucocorticoid stimulation was also associated with an increase in neural excitability, which could in fact improve the detection of sensory information [9]. Additionally, proopiomelanocortin (POMC), an anterior pituitary hormone, is synthetized secondary to decreased glucocorticoid and produces higher endorphin levels. Higher endorphin levels are correlated with hallucinations and psychosis [9].

Our case report has some limitations. First, we could not define if the patient started with a true severe depressive disorder, which led to psychotic symptoms, or if the depressive symptoms were negative manifestations of a primary psychotic disorder. We are prone to the latter diagnosis because of symptom severity, the presence of visual hallucinations, the severe thinking impairment, persistent echolalia, and the complex speech disorder. Second, it was not possible to measure renin activity, aldosterone, or adrenal androgens due to laboratorial limitations. Finally, we present a rare manifestation of adrenal insufficiency that highlights the importance of the glucocorticoid metabolism to cognitive and behavioral functions and the need to better understand the etiology of psychiatric manifestations of adrenal insufficiency.

## Figures and Tables

**Table 1:** Laboratorial data.

Variable	Admission	3rd day	6th day	13th day	20th day

Hemoglobin (g/dL)	15.0	12.9	11.6	12.7	13.0
Hematocrit (%)	43.7	37.2	35.3	39.8	40.8
Mean corpuscular volume (*μ*m^3^)	76.4	78.3	79.8	83.6	85.5
Platelets (per mm^3^)	215.000	160.000	221.000	280.000	312.000
White-cell count (per mm^3^)	8.100	4.850	6.030	11.060	9.150
Differential count (%)					
*Neutrophils *	0.462	0.528	0.7	0.504	0.82
*Eosinophils *	0.035	0.075	0.003	0.041	0
*Lymphocytes *	0.379	0.299	0.214	0.382	0.15
*Monocytes *	0.12	0.096	0.082	0.071	0.03
Urea (mg/dL)	41.0	25.1	19.7	30.1	31.9
Creatinine (mg/dL)	0.8	0.6	0.6	0.7	0.7
Sodium (mmol/L)	111	109	132	135	135
Potassium (mmol/L)	5.8	4.3	3.9	3.0	3.3
Iron (*μ*g/dL)			25		
Ferritin (ng/mL)			361.1		
Transferrin saturation (%)			16.3		
Vitamin B12 (pg/mL)			864		
Folic acid (ng/mL)			4.71		
C-reactive protein (mg/L)	18.0	17.3		1.0	2.4
Thyroid-stimulating hormone (*μ*IU/mL)			1.87		
Free thyroxine (ng/dL)			1.18		
Glucose (mg/dL)		91		88	
Aspartate transaminase (U/L)			27		
Alanine transaminase (U/L)			28		
Adrenocorticotropic hormone (pg/mL)			364		
Cortisol (*μ*g/dL)			4.7		
Urinary cortisol (*μ*g/24 h)			9.540		
